# Upper Gastrointestinal Mucosal Damage and Subsequent Risk of Parkinson Disease

**DOI:** 10.1001/jamanetworkopen.2024.31949

**Published:** 2024-09-05

**Authors:** Jocelyn J. Chang, Subhash Kulkarni, Trisha S. Pasricha

**Affiliations:** 1 Tufts University School of Medicine, Boston, Massachusetts; 2 Division of Gastroenterology, Department of Medicine, Beth Israel Deaconess Medical Center, Boston, Massachusetts; 3 Harvard Medical School, Boston, Massachusetts

## Abstract

**Importance:**

The gut-first hypothesis of Parkinson disease (PD) has gained traction, yet potential inciting events triggering Parkinson pathology from gut-related factors remain unclear. While *Helicobacter pylori* infection is linked to mucosal damage (MD) and PD, it is unknown how upper gastrointestinal MD from any source increases PD risk.

**Objective:**

To evaluate any association between upper endoscopy findings of MD and subsequent clinical PD diagnosis.

**Design, Setting, and Participants:**

This was a retrospective cohort study of patients with no PD history undergoing upper endoscopy with biopsy between January 2000 and December 2005, with final follow-up assessments completed July 31, 2023. The study was conducted within the Mass General Brigham system, a multicenter network in the greater Boston, Massachusetts, area. Patients with MD were matched 1:3 to patients without MD based on age, sex, and date of initial endoscopy.

**Exposure:**

MD, defined as erosions, esophagitis, ulcers, or peptic injury, observed on upper endoscopy or pathology reports.

**Main Outcomes and Measures:**

The relative risk of PD given a history of MD, estimated using incident rate ratio (IRR) and multivariate Cox proportional hazard ratios (HRs).

**Results:**

Of 9350 patients, participants had a mean (SD) age of 52.3 (20.3) years; 5177 (55.4%) were male; and 269 (2.9%) were Asian, 737 (7.9%) Black, and 6888 (73.7%) White. Most participants underwent endoscopy between the ages of 50 and 64 years (2842 [30.4%]). At baseline, patients with MD were more likely to have a history of *H pylori* infection, proton-pump inhibitor use, chronic nonsteroidal anti-inflammatory drug use, gastroesophageal reflux disease, smoking, constipation, and dysphagia. The mean (SD) follow-up time was 14.9 (6.9) years for the whole cohort, during which patients with MD were more likely to develop PD (IRR, 4.15; 95% CI, 2.89-5.97; *P* < .001) than those without MD, even after covariate adjustment (HR, 1.76; 95% CI 1.11-2.51; *P* = .01). Constipation, dysphagia, older age, and higher Charlson-Deyo Comorbidity Index were also associated with higher PD risk.

**Conclusions and Relevance:**

In this cohort study, a history of upper gastrointestinal MD was associated with elevated risk of developing a clinical PD diagnosis. Increased vigilance among patients with MD for future PD risk may be warranted.

## Key Points

**Question:**

Is upper gastrointestinal mucosal damage associated with increased risk of subsequent Parkinson disease (PD) diagnosis?

**Findings:**

In this cohort study of 9350 patients with no prior history of PD, findings of mucosal damage on upper endoscopy were associated with a 76% greater risk of developing a clinical PD diagnosis.

**Meaning:**

These findings suggest that increased vigilance among patients with upper gastrointestinal mucosal damage for future PD risk may be warranted.

## Teaser

This cohort study evaluates whether mucosal damage, observed on upper endoscopy, is associated with subsequent diagnosis of Parkinson disease.

## Introduction

Parkinson disease (PD), a progressive neurodegenerative disorder, affects 8.5 million people worldwide based on 2019 estimates,^1^ with prevalence more than doubling over the past 3 decades. Characterized by the degeneration of dopaminergic neurons in the substantia nigra, PD manifests through motor symptoms such as tremors, rigidity, and bradykinesia as well as nonmotor symptoms including constipation.

The Braak hypothesis, rooted in neuropathological staging of PD, outlines a consistent progression pattern from specific induction sites.^2^ Additionally, accumulating evidence indicates a prolonged prodromal phase of PD, lasting years and characterized by significant gastrointestinal (GI) dysfunction.^3^ The convergence of these factors suggests a gut-first hypothesis, proposing that in a subset of individuals, pathology may originate in the gut and travel to the brain through the vagus nerve. Recent animal studies reinforce this concept, showing the transmission of gut-initiated pathology via the vagus nerve, leading to central nervous system neurodegeneration and motor deficits.^4,5^ While many patients’ disease may well originate within the brain or possibly involve an olfactory mechanism, these findings provide substantial support for one pathway of PD pathogenesis that originates in the gut. However, the precise inciting factor that may trigger such pathology remains elusive, complicating efforts to fully understand PD’s onset in this proposed subset of gut-first individuals. A recent nationwide study found no association between a history of inflammatory bowel disease and subsequent risk of PD, suggesting that mucosal damage (MD) in the distal GI tract where pelvic rather than vagal innervation is the hallmark may play a less important role in pathogenesis.^6^

In this context, *Helicobacter pylori* infection, a well-known risk factor for upper GI tract ulcers and malignant neoplasms, has emerged as a point of interest. Epidemiological studies have reported a higher prevalence of *H pylori* infection in patients with PD compared with the general population, implying a possible association between *H pylori* and PD.^7,8,9^ The pathogen’s role in inducing upper GI MD and its systemic inflammatory responses present a compelling area for investigation in relation to PD.

Indeed, a significant gap remains in understanding the relationship between broader upper GI MD, such as peptic ulcer disease (PUD) and erosions, and the development of PD, as most studies to date have focused on correlational links to a history of *H pylori* infection. Additionally, while nonsteroidal anti-inflammatory drug (NSAID) use is a known risk factor for PUD, existing literature is mixed regarding its role in PD. Some studies have suggested a possible neuroprotective effect of NSAIDs^10^ and an associated reduced risk of PD, while other studies^11,12^ found no evidence for association. We hypothesized that significant pathological defects to GI mucosa, including but not limited to those associated with *H pylori* and NSAID use, may be associated with subsequent development of PD—although the net effect of NSAIDs on PD risk might be offset by its proposed neuroprotective properties.

Our study explores this gap by examining the association between upper GI MD, confirmed through upper endoscopy (EGD) findings, and the subsequent development of a clinical PD diagnosis. By retrospectively analyzing a cohort of patients who underwent EGD, this study aims to develop a better understanding of the potential gut-first pathway of PD pathogenesis, thereby contributing to a more comprehensive understanding of the disease and opening new avenues for early intervention and treatment strategies.

## Methods

### Retrospective Cohort Study Design

We performed a retrospective cohort study to determine the risk of developing PD among patients with a history of MD compared with those without. We then performed a nested case-control study to assess for specific covariates that might account for the associations observed in the broader cohort. The study protocol was approved by the Massachusetts General Hospital institutional review board, which granted an exemption of informed consent because all data were fully deidentified and this study was considered a secondary analysis of existing data. This study followed the Strengthening the reporting of Observational Studies in Epidemiology (STROBE) reporting guideline.

### Patient Selection and Follow-Up

Patients were selected using Research Patient Data Registry (RPDR), a Mass General Brigham (MGB)–based electronic database that stores data on patient demographics, encounters, medications, procedures, and billing codes. The registry encompasses a representation of urban academic centers as well as satellite outpatient clinics and community hospitals in the greater Boston area. Using the RPDR search query tool, we identified a cohort of 18 305 patients based on the following inclusion criteria: (1) within the MGB system, (2) history of EGD with biopsy between 2000 and 2005, and (3) no history of PD (*International Statistical Classification of Diseases and Related Health Problems, Tenth Revision *[ICD-10] code G20) prior to initial EGD.

Patients with positive endoscopic findings for MD were matched with patients without MD in a 1:3 ratio based on age, sex, and date of EGD. MD was defined as the presence of *erosion*, *esophagitis*, *ulcer*, or *peptic injury* observed on EGD or pathology reports. Final cohort size after matching is shown in Table 1. Each participant was followed up until (1) diagnosis of PD; (2) death; or (3) in the absence of a PD diagnosis, censoring at an outpatient appointment with no subsequent follow-up for more than 2 years, representing loss to follow-up, or until July 31, 2023, the date of final follow-up assessments.

**Table 1.** Patient Characteristics^a^

Characteristic	Patients, No. (%)			*P* value
	With MD (n = 2337)	Without MD (n = 7013)	Total (N = 9350)	
Demographic characteristics				
Age at endoscopy, mean (SD), y	52.2 (18.6)	52.3 (20.8)	52.3 (20.3)	.84
Age group at endoscopy, y				
<18	161 (6.9)	616 (8.8)	777 (8.3)	<.001
18-29	132 (5.6)	410 (5.8)	542 (5.8)	
30-49	619 (26.5)	1744 (24.9)	2363 (25.3)	
50-64	798 (34.1)	2044 (29.1)	2842 (30.4)	
≥65	627 (26.8)	2199 (31.4)	2826 (30.2)	
Sex				
Female	1038 (44.4)	3135 (44.7)	4173 (44.6)	.81
Male	1299 (55.6)	3878 (55.3)	5177 (55.4)	
Race				
Asian	57 (2.4)	212 (3.0)	269 (2.9)	.02
Black	173 (7.4)	564 (8.0)	737 (7.9)	
White	1807 (77.3)	5081 (72.5)	6888 (73.7)	
Other^b^	177 (7.6)	177 (6.9)	664 (7.1)	
Unknown^c^	123 (5.3)	669 (9.5)	792 (8.5)	
Covariates				
Constipation	996 (42.6)	1606 (22.9)	2602 (27.8)	<.001
Dysphagia	984 (42.1)	1887 (26.9)	2871 (30.7)	<.001
*H pylori* infection	169 (7.2)	231 (3.3)	400 (4.3)	<.001
CCI, mean (SD)	0.57 (1.34)	0.39 (1.11)	0.43 (1.57)	<.001

Abbreviations: CCI, Charlson-Deyo Comorbidity Index; *H pylori*, *Helicobacter pylori*; MD, mucosal damage.

^a^ Patients were matched 1:3 (with MD to without MD) by age, sex, and initial upper endoscopy date.

^b^ Identified as not fitting into other categories with no further definition.

^c^ Patients who declined disclosing race.

### Outcomes and Exposures

Cases of PD were identified through a *International Classification of Diseases, Ninth Revision *(*ICD-9*) code 332.0 and *ICD-10* code G20 screen that was confirmed by a history of PD-specific prescriptions, of which levodopa-based preparations are the mainstay of treatment. Comparable identification algorithms for PD cases have been shown to have positive predictive values of 88% to 90%.^13,14^

To identify comorbidities, we used the enhanced version of Charlson-Deyo Comorbidity Index (CCI) for administrative data. Other covariates were chosen based on their known or suspected links to MD or PD, as suggested by existing literature.^3,15,16^ In particular, dysphagia and constipation, which have been associated with an increased risk of subsequent new-onset diagnosis of idiopathic PD,^6^ as well as *H pylori*, which is associated with an increased risk of gastrointestinal MD, were included as covariates.

Age, sex, race, and body mass index (BMI; calculated as weight in kilograms divided by height in meters squared) were extrapolated from demographic data in the electronic health record. Racial categories were Asian, Black, White, other (identified as not fitting into other categories with no further definition), and unknown. Regarding medications, long-term use of NSAIDs was identified using *ICD-9* code V58.64 and *ICD-10 *code Z79.1; and proton-pump inhibitor (PPI) use, dichotomized as any history or none, was extracted from medication prescription history. For substance use history, alcohol use disorder was defined by *ICD-9* codes 303X and 305.0X and *ICD-10* equivalent F10X; history of complications related to alcohol use, including alcoholic gastritis and alcoholic liver disease, were defined by *ICD-9* codes 202, 535.3, 525.3, 571X, and 980, along with *ICD-10* equivalents K29.2 and K70X; and chronic tobacco smoking history or exposure was identified through *ICD-9* codes 305.1X and E869.4 and *ICD-10* equivalents Z72.0, Z87.891, Z57.31, and Z77.22. For GI covariates, history of constipation, dysphagia, and gastroesophageal reflux disease (GERD) were defined per *ICD-9* codes 564.0X, 787.2X, 530.11, and 530.81, respectively, in addition to *ICD-10*-equivalents K59.0X, R13.1X, and K21X, respectively; *H pylori* infection was screened for per *ICD-9* code 41.86 and *ICD-10* code B96.81 and then confirmed by *H pylori* identified on endoscopic pathology stains.

### Nested Case-Control Studies

Building on our cohort study, 2 nested case-control analyses were conducted: one within the group of patients previously found without MD and the second within the group of patients previously found to have MD. Specific factors were assessed related to MD that might account for any associations observed in the broader cohort. In this way, we sought to clarify factors that may be of particular relevance to a putative gut-first trigger of PD.

### Patient Selection and Covariates for Nested Case-Control Studies

In the first nested cohort, we defined cases as patients without MD subsequently diagnosed with PD. Controls were defined as patients without MD who did not later develop PD according to follow-up. In the second nested cohort, we defined cases as patients with MD subsequently diagnosed with PD. Controls were defined as patients with MD who did not later develop PD according to follow-up. In both nested analyses, cases were matched with controls in a 1:2 ratio based on age at EGD, sex, and date of each case’s PD diagnosis by incidence density sampling. Covariates were determined in the same manner as in the retrospective study.

### Statistical Analysis

Based on patient outcomes and exposures, descriptive characteristics of our cohort were reported as means and SDs for continuous variables, and total number and percentage of total number for categorical variables. Descriptive data were then analyzed using *t* tests and χ^2^ tests or Fisher exact tests, respectively, with *P* ≤ .05 considered statistically significant. Data analysis was conducted in R version 4.3.1 (R Project for Statistical Computing).

Incidence rate of PD in our matched cohort was calculated per 10 000 person-years and compared with the incidence of PD in the general US population for generalizability. We then calculated the incidence rate ratio (IRR) of PD in patients with MD vs patients without MD.

Multivariate Cox proportional-hazards analysis was conducted in a stepwise manner, with *P* ≤ .05 considered statistically significant, to incrementally assess the association between each covariate and the risk of developing PD, ensuring a thorough examination of their individual and combined effects without overfitting our model. We then assessed for possible violation of the proportional hazards assumption with log-log plots and Schoenfeld residuals, confirming that the assumption in our Cox model was not violated.

For the nested case-control studies, crude and adjusted odds ratios with 95% CIs were calculated using conditional logistic regression to determine the association between PD and specific covariates. The covariates were chronic smoking, esophagitis, *H pylori*, chronic NSAID use, PPI use, and GERD.

## Results

**Figure 1.**
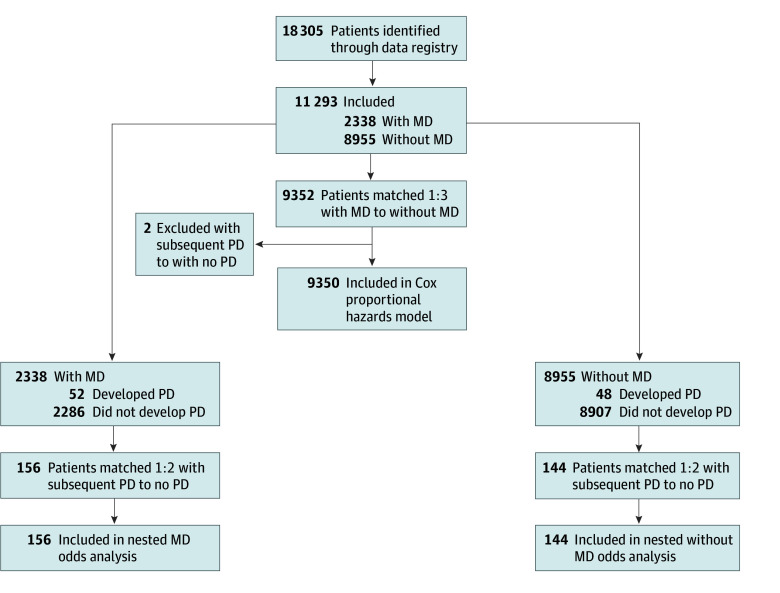
Study Flowchart MD indicates mucosal damage; PD, Parkinson disease.

The total cohort size was 9350 patients (Figure 1). Overall, 269 participants (2.9%) were Asian, 737 (7.9%) Black, 6888 (73.7%) White, and 664 (7.1%) who identified as another race. The majority were male (5177 [55.4%]) and between 50 and 64 years of age (2842 [30.4%]) or 65 years and older (2826 [30.2%]), with a mean (SD) age at endoscopy of 52.3 (20.3) years. Overall, 2337 patients were found to have evidence of MD on endoscopy while 7013 did not. Differences in prevalence of covariates are summarized in Table 1.

**Figure 2.**
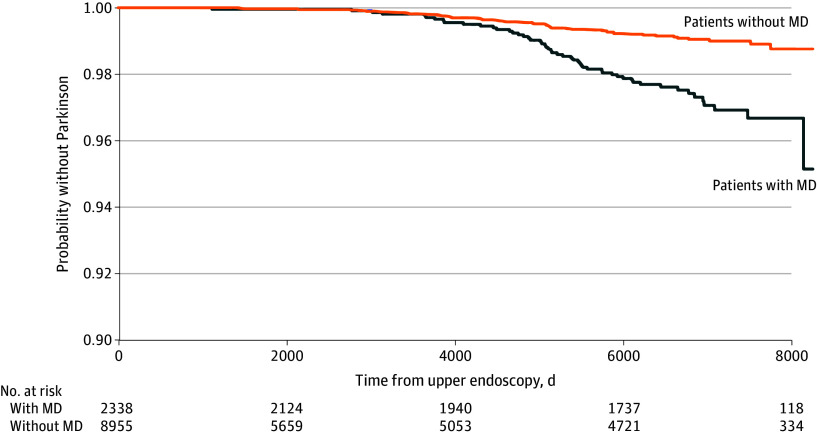
Survival Curves for Time to Parkinson Disease Diagnosis MD indicates mucosal damage.

Of the 2338 patients with MD, 52 patients (2.2%) were later diagnosed with PD, while of the 8955 patients without MD, 48 (0.5%) were later diagnosed with PD. In other words, of the 100 patients who were diagnosed with PD in this cohort, 52 had MD on initial biopsy (52%). These results indicate a notably higher likelihood of PD diagnosis among patients with MD compared with those without MD (prevalence, 2.2% vs 0.5%; *P* < .001; IRR, 4.15; 95% CI, 2.89-5.97; *P* < .001). After adjusting for additional covariates in the Cox model (age at endoscopy, sex, race, CCI, constipation, dysphagia, and *H pylori*), the risk associated with MD remained pronounced (hazard ratio [HR], 1.76; 95% CI, 1.11-2.51; *P* = .01). Overall incidence of PD in our cohort was 16 per 100 000 person-years, comparable with reported overall incidence of 17 per 100 000 person-years worldwide.^17^ Survival curves for time to PD diagnosis was less favorable for patients with MD than those without (Figure 2). Mean (SD) lead-time between detection of MD and PD diagnosis was 14.2 (3.5) years, and mean (SD) age at PD diagnosis was 72.7 (12.2) years in our whole cohort.

**Figure 3.**
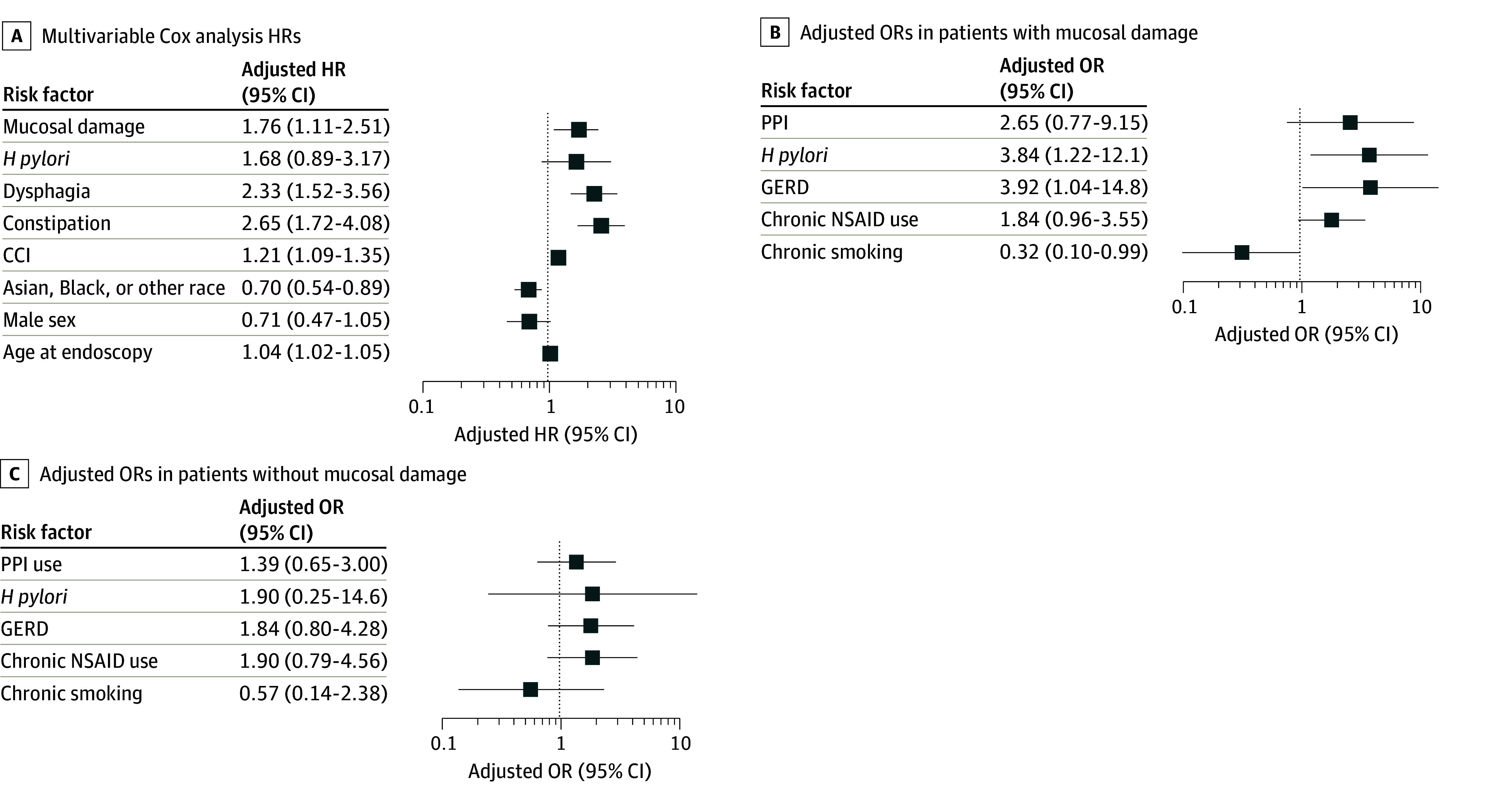
Association of Risk Factors With Parkinson Disease Diagnosis Other race and ethnicity included Asian and Black individuals as well as those with racial identifications that did not fit into those categories. CCI indicates Charlson-Deyo Comorbidity Index; GERD, gastroesophageal reflux disease; *H pylori*, *Helicobacter pylori*; HR, hazard ratio; NSAID, nonsteroidal anti-inflammatory drug; OR, odds ratio; PPI, proton-pump inhibitor.

Factors associated with an increased risk of developing PD included age (HR, 1.04; 95% CI, 1.02-1.05; *P* < .001), CCI (HR, 1.21; 95% CI, 1.09-1.35; *P* < .001), constipation (HR, 2.65; 95% CI, 1.72–4.08; *P* < .001), and dysphagia (HR, 2.33; 95% CI, 1.52-3.56; *P* < .001). Asian, Black, and other race were associated with a decreased risk of developing PD (HR, 0.70; 95% CI, 0.54-0.89; *P* = .004), Sex and history of *H pylori* infection on endoscopy were not associated with risk of PD (Table 2 and Figure 3A).

**Table 2.** Variables Associated With PD Development in Patients With Upper Endoscopy Biopsy

Variable	Result (95% CI)	*P* value
**Multivariate hazard ratios from Cox proportional hazard model (n = 9350; patients with PD = 100)**		
Age at endoscopy	1.04 (1.02-1.05)	<.001
Sex		
Female	1 [Reference]	.09
Male	0.71 (0.47-1.05)	
Race		
White	1 [Reference]	.004
Other race and ethnicity^a^	0.70 (0.54-0.89)	
CCI^b^	1.21 (1.09-1.35)	<.001
Constipation	2.65 (1.72-4.08)	<.001
Dysphagia	2.33 (1.52-3.56)	<.001
*H pylori*	1.68 (0.89-3.17)	.11
MD	1.76 (1.11-2.51)	.01
**Nested odds ratios in patients with MD on initial biopsy (n = 156; patients with PD = 52)** ^c^		
Chronic smoking	0.32 (0.10-0.99)	.05
Chronic NSAID use	1.84 (0.96-3.55)	.07
GERD	3.92 (1.04-14.76)	.04
*H pylori*	3.84 (1.22-12.13)	.02
PPI	2.65 (0.77-9.15)	.12
**Nested odds ratios in patients without MD on initial biopsy (n = 144; patients with PD = 48)** ^c^		
Chronic smoking	0.57 (0.14-2.38)	.44
Chronic NSAID use	1.90 (0.79-4.56)	.15
GERD	1.84 (0.80-4.28)	.15
*H pylori*	1.90 (0.25-14.6)	.54
PPI	1.39 (0.65-3.00)	.40

Abbreviations: CCI, Charlson-Deyo Comorbidity Index; GERD, gastroesophageal reflux disease; *H pylori*, *Helicobacter pylori*; MD, mucosal damage; NSAID, nonsteroidal anti-inflammatory drug; PD, Parkinson Disease; PPI, proton-pump inhibitor.

^a^ Other race and ethnicity included Asian and Black individuals as well as those with racial identifications that did not fit into those categories.

^b^ Where each additional point in the CCI score represents a 21% increase in the risk of developing PD compared with baseline risk.

^c^ Matched by age at upper endoscopy, sex, and date of cases’ PD diagnosis.

Within our first nested subgroup of patients with MD, while no significant demographic differences were noted between patients with a clinical diagnosis PD and those without, those with PD had a higher baseline prevalence of chronic NSAID use and GERD compared with patients who did not develop PD (eTable 1 in Supplement 1). The following covariates were incorporated into the analysis: chronic NSAID use, chronic smoking, GERD, PPI use, and *H pylori* (Table 2 and Figure 3B). Presence of *H pylori* on initial biopsy was associated with a markedly higher probability of developing a diagnosis of PD, as indicated by an adjusted odds ratio (aOR) of 3.84 (95% CI, 1.22-12.13; *P* = .02). GERD was also associated with increased PD diagnosis risk (aOR, 3.92; 95% CI, 1.04-14.76; *P* = .04). Chronic smoking, chronic NSAID use, and PPI use were not associated with PD risk.

Within our second nested subgroup of patients without MD, similarly no significant demographic differences were noted between patients with a clinical diagnosis of PD and those without. Patients with PD had a higher baseline prevalence of GERD compared with patients who did not develop PD (eTable 2 in Supplement 1). As in the first nested case-control analysis, the following covariates were incorporated into this analysis: chronic NSAID use, chronic smoking, GERD, PPI use, and *H pylori* (Table 2 and Figure 3C). However, none of these covariates were associated with an increased risk of developing a diagnosis of PD in this subgroup analysis of patients without MD.

## Discussion

The findings of our investigation corroborate our hypothesis that upper gastrointestinal MD would be associated with clinical PD development, reinforcing the theory of a gut-first progression in PD in a subset of patients. These findings likely suggest 1 of 2 possibilities: first, that MD may serve as an inciting event that could precipitate pathologic alpha-synuclein misfolding in the gut. Second, as dopamine is known to play a key gastroprotective role,^18^ it may be that patients with subclinical dopaminergic signaling reduction are at higher risk of MD and that alpha-synuclein pathology preceded this event. Understanding these mechanisms is of great interest in future research endeavors.

An important strength of our study is its access to endoscopic reports and pathologic diagnoses to establish MD and history of *H pylori* infection, not merely relying on *ICD* codes. Our comprehensive analysis indicates a marked escalation in PD risk among individuals with MD (IRR, 4.15; 95% CI, 2.89-5.97; *P* < .001). This association persisted even after adjustment for established covariates (HR, 1.76; 95% CI, 1.11-2.51; *P* = .01), underpinning the pivotal role of the gut-brain axis in neurodegenerative conditions and highlighting GI factors as potential early biomarkers and contributors to PD pathogenesis. The substantial 14.2-year mean lead-time between detection of MD and diagnosis of PD likewise strengthens the hypothesis that MD precedes motor symptoms of PD.

Many of our findings are consistent with existing literature, which identifies older age, White race, constipation, and dysphagia as risk factors for PD.^3^ Additionally, consistent with prior studies^19^ that show increased prevalence of GERD in patients with PD, our investigation reveals a noteworthy positive association between GERD and PD. At baseline within our first nested analysis of patients with MD, prevalence of GERD was significantly higher in patients with PD than those without PD (eTable 1 in Supplement 1). GERD continued to demonstrate a positive association with PD in the context of a history of MD (aOR, 3.92; 95% CI, 1.04-14.76; *P* = .04) (Table 2). PPI usage, aimed at mitigating MD related to GERD, had an aOR greater than 1, but this was not statistically significant in our analysis. Of note, there are limitations in utilizing *ICD* codes to quantify PPI use, which capture neither duration nor dosage of medication use, and such limitations may explain the lack of association with PD.

Furthermore, our research observed a complex interplay between *H pylori* infection, MD, and the risk of PD. The initial presence of *H pylori* noted on biopsies was associated with an increased probability of PD (aOR, 3.84; 1.22-12.13; *P* = .02) within the context of MD, but not among those without MD (aOR, 1.90; 95% CI, 0.25-14.6; *P* = .54), indicating a multifaceted interaction in which *H pylori*’s role in PD risk amplification may be contingent on concurrent MD. Indeed, there was no significant difference in *H pylori* infection rates between patients with MD and those without MD in our cohort (Table 1). While prevalence of *H pylori* infection worldwide is approximately 50%, with rates ranging from 35% to 40% in the United States, only approximately 20% of infected individuals develop related gastroduodenal disorders, including PUD, during their lifetime; individuals with *H pylori* infection often present asymptomatically.^20,21^

In the context of MD, there was no association between chronic use of NSAIDs and risk for PD, although the aOR was greater than 1. While NSAIDs have been well-established to cause MD through a variety of mechanisms,^22^ existing literature is equivocal regarding the role of NSAIDs in modifying PD risk. Some studies cite evidence of reduced PD risk with NSAID use,^23,24^ while other studies indicate no such benefit.^11^ How these possibly conflicting effects may impact PD in the context of prior MD cannot be definitively evaluated in the present study, as we lacked sufficient information on the indication for NSAID use, and therefore cannot exclude the possibility of confounding by indication given potential links between cardiovascular or inflammatory conditions and PD.^25^

Similarly, a history of chronic smoking had an aOR of less than 1 among those with MD, although the finding was not statistically significant. While smoking is a well-established risk factor for PUD, other studies have suggested a protective, dose-dependent inverse association between tobacco smoking and PD risk.^26,27,28,29^ Nicotine has been shown experimentally to prevent parkinsonism in rodents and induce brain-derived neurotrophic factor in the striatum,^30^ thereby appearing to be neuroprotective for dopaminergic neurons. It remains unclear whether smoking may have a sufficient countering effect in PD-specific gut pathology.

Overall, given the apparent differences in results between the 2 nested case-control analyses, our data suggest that the association between MD and PD risk may not be contingent on one isolated abnormality but rather a cumulative effect of multiple GI inflammatory insults. These observations suggest that the cumulative burden or the diversity of inflammation sites within the GI tract could be instrumental in PD risk elevation, possibly through a threshold effect on the gut-brain axis.

### Limitations

This study has several limitations. First, we were not able to capture cases of PD that were diagnosed and treated outside the MGB system—and indeed of MD that may have occurred outside our system. This presents a risk of a carry-over effect if, for example, a diagnosis of PD was made before the patient entered our cohort but was not mentioned until after experiencing MD. We believe that this may be mitigated by our efforts to confirm a diagnosis of PD through assessing prescription information. Additionally, given our large cohort, we were able to identify an incidence of PD comparable with the general population, so it is likely that we closely approximated the true population burden. Second, differences existed in baseline age distribution and mean CCI between patients with MD compared with those without. We believe that bias introduced from these baseline differences were mitigated by including both age and CCI as covariates in our Cox proportional hazards model. However, surveillance bias toward patients with MD remains a possibility. Third, outside of MD defined per endoscopic and pathologic reports, we relied on *ICD* codes for several exposures and outcomes. Fourth, the sample sizes in our nested case-control analyses were relatively small and so additional confirmatory studies should be done in the future before drawing firm conclusions, particularly regarding a lack of association, in these results. Additionally, although we addressed several possible confounders in our analysis, as a retrospectively conducted cohort study and nested case-control study, there is risk of bias from unknown confounding.

## Conclusions

This study found that a history of upper GI MD was associated with an increased risk of subsequently developing PD. These findings highlight the necessity for heightened monitoring of patients with MD given their increased clinical PD susceptibility and the importance of establishing gut biomarkers. With PUD globally affecting upwards of 8.09 million people^31^ and *H pylori* infection even more widespread, timely detection and treatment of *H pylori* infection, along with MD management, may prove crucial to early recognition of risk of and potentially intervention against PD.

## Notes

Supplement 1. **eTable 1.** Nested Patients With MD Cohort Characteristics **eTable 2.** Nested Patients Without MD Cohort Characteristics

Supplement 2.
Data Sharing Statement
