# Supplementary material for: Upper Gastrointestinal Mucosal Damage and Subsequent Risk of Parkinson Disease
Source: JAMA Netw Open. 2024 Sep 5;7(9):e2431949. doi: 10.1001/jamanetworkopen.2024.31949 (PMC11378005; doi:10.1001/jamanetworkopen.2024.31949)
Supplement: Supplement 1. — eTable 1. Nested Patients With MD Cohort Characteristics eTable 2. Nested Patients Without MD Cohort Characteristics [file jamanetwopen-e2431949-s001.pdf]

Supplemental Online Content

Chang JJ, Kulkarni S, Pasricha TS. Upper gastrointestinal mucosal damage and subsequent risk of Parkinson disease. *JAMA Netw Open.* 2024;7(9):e2431949. doi:10.1001/jamanetworkopen.2024.31949

eTable 1. Nested Patients With MD Cohort Characteristics

eTable 2. Nested Patients Without MD Cohort Characteristics

This supplemental material has been provided by the authors to give readers additional information about their work.

© 2024 Chang JJ et al. *JAMA Network Open.*

eTable 1. Nested Patients With MD Cohort Characteristics				
matched 1:2 by age, sex, initial endoscopy date[a]				
Characteristic	Patients with PD (n=52)	Patients without PD (n=104)	Total (n=156)	p-value
**Demographics**				
Mean age at endoscopy (SD)	58.8 (12.7)	59.2 (13.6)	59.1 (13.3)	0.86
**Age at endoscopy**, years				0.28
<18	1 (1.9)	0	1 (0.6)	
18-29	0	2 (1.9)	2 (1.3)	
30-49	7 (13.5)	22 (21.2)	29 (18.6)	
50-64	28 (53.8)	43 (41.3)	71 (45.5)	
≥65	16 (30.8)	37 (35.6)	53 (34.0)	
**Sex**				
Female	29 (55.8)	59 (56.7)	88 (56.4)	0.91
Male	23 (44.2)	45 (43.3)	68 (43.6)	
**Race**				0.77
Asian	1 (1.9)	3 (2.9)	4 (2.6)	
Black	3 (5.8)	10 (9.6)	13 (8.3)	
White	44 (84.6)	78 (75.0)	122 (78.2)	
Other[b]	4 (7.7)	11 (10.6)	15 (9.6)	
Unknown[c]	0	2 (1.9)	2 (1.3)	
**Medical history**				
Chronic NSAIDs	22 (42.3)	25 (24.0)	47 (30.1)	0.02
Chronic smoking	5 (9.6)	20 (19.2)	25 (16.0)	0.12
Constipation	38 (73.1)	42 (40.4)	80 (51.3)	<0.001
Dysphagia	36 (69.2)	40 (38.5)	76 (48.7)	<0.001
GERD	49 (94.2)	82 (78.8)	131 (84.0)	0.014
H. pylori infection	8 (15.4)	8 (7.7)	16 (10.3)	0.14
PPI use	48 (92.3)	84 (80.8)	132 (84.6)	0.06
Mean CCI (SD)	1.04 (1.87)	0.66 (1.24)	0.79 (1.48)	0.13

PD, Parkinson's Disease; NSAID, non-steroidal anti-inflammatory drug; GERD, gastroesophageal reflux disease; H pylori, Helicobacter pylori; PPI, proton pump inhibitor; CCI, Charlson Comorbidity Index

[a]Values are presented as No. (%) patients unless otherwise noted

[b]Identified as not fitting into other categories

[c]Patients who declined disclosing race

© 2024 Chang JJ et al. *JAMA Network Open.*

eTable 2. Nested Patients Without MD Cohort Characteristics

matched 1:2 by age, sex, initial endoscopy date[a]

Characteristic	Patients with PD (n=48)	Patients without PD (n=96)	Total (n=144)	p-value
**Demographics**				
Mean age at endoscopy (SD)	58.8 (11.7)	58.6 (17.2)	58.7 (15.5)	0.94
**Age at endoscopy**, years				0.13
<18	1 (2.1)	2 (2.1)	3 (2.1)	
18-29	0 (0)	6 (6.3)	6 (4.2)	
30-49	6 (12.5)	17 (17.7)	23 (16.0)	
50-64	26 (54.2)	33 (34.4)	59 (41.0)	
≥65	15 (31.3)	38 (39.6)	53 (36.8)	
**Sex**				
Female	17 (35.4)	34 (35.4)	51 (35.4)	1.00
Male	31 (64.6)	62 (64.6)	93 (64.6)	
**Race**				0.08
Asian	0 (0)	1 (1.0)	1 (0.7)	
Black	3 (6.3)	8 (8.3)	11 (7.6)	
White	43 (89.6)	67 (69.8)	110 (76.4)	
Other[b]	1 (2.1)	7 (7.3)	8 (5.6)	
Unknown[c]	1 (2.1)	13 (13.5)	14 (9.7)	
**Medical history**				
Chronic NSAIDs	7 (14.6)	6 (6.3)	13 (9.0)	0.10
Chronic smoking	3 (6.3)	9 (9.4)	12 (8.3)	0.53
Constipation	24 (50.0)	24 (25.0)	48 (33.3)	0.003
Dysphagia	27 (56.3)	25 (26.0)	52 (36.1)	<0.001
GERD	37 (77.1)	57 (59.4)	94 (65.3)	0.04
H. pylori infection	2 (4.2)	2 (2.1)	4 (2.8)	0.47
PPI use	30 (62.5)	47 (49.0)	77 (53.5)	0.13
Mean CCI (SD)	0.8 (1.7)	0.5 (1.2)	0.6 (1.4)	0.28

PD, Parkinson's Disease; NSAID, non-steroidal anti-inflammatory drug; GERD, gastroesophageal reflux disease; H pylori, Helicobacter pylori; PPI, proton pump inhibitor; CCI, Charlson Comorbidity Index

[a]Values are presented as No. (%) patients unless otherwise noted

[b]Identified as not fitting into other categories

[c]Patients who declined disclosing race

© 2024 Chang JJ et al. *JAMA Network Open.*
