# Supplementary material for: Upper Gastrointestinal Mucosal Damage and Subsequent Risk of Parkinson Disease
Source: JAMA Netw Open. 2024 Sep 5;7(9):e2431949. doi: 10.1001/jamanetworkopen.2024.31949 (PMC11378005; doi:10.1001/jamanetworkopen.2024.31949)
Supplement: Supplement 2. — Data Sharing Statement [file jamanetwopen-e2431949-s002.pdf]

Data Sharing Statement

Chang. Upper Gastrointestinal Mucosal Damage and Subsequent Risk of Parkinson Disease. *JAMA Netw Open*. Published September 05, 2024. doi:10.1001/jamanetworkopen.2024.31949

Data
Data available: Yes
Data types: Deidentified participant data
How to access data: Deidentified participant data and analytic code are available upon email request (tpasrich@bidmc.harvard.edu).
When available: With publication

Supporting Documents
Document types: Statistical/analytic code
How to access documents: as above--"Deidentified participant data and analytic code are available upon email request (tpasrich@bidmc.harvard.edu)."
When available: With publication

Additional Information
Who can access the data: x
Types of analyses: x
Mechanisms of data availability: x
Any additional restrictions: x
